# Cefiderocol Susceptibility in Japanese Clinical Enterobacterales Isolates and the Effect of IMP-Type Carbapenemases on Resistance

**DOI:** 10.3390/antibiotics15070667

**Published:** 2026-07-08

**Authors:** Manke Cai, Go Yamamoto, Shigeto Hamaguchi, Akiko Ueda, Ryuji Kawahara, Daisuke Motooka, Yusuke Takahashi, Satoshi Kutsuna

**Affiliations:** 1 Department of Infection Control and Prevention, Graduate School of Medicine, The University of Osaka, 2-2 Yamadaoka, Suita, Osaka 565-0871, Japan; 2 Department of Transformative Protection to Infectious Disease, Graduate School of Medicine, The University of Osaka, 2-2 Yamadaoka, Suita, Osaka 565-0871, Japan; 3 Osaka Institute of Public Health, 1-3-3 Nakamichi, Higashinari-ku, Osaka 537-0025, Japan; 4 Department of Clinical Laboratory, The University of Osaka Hospital, 2-15 Yamadaoka, Suita, Osaka 565-0871, Japan; 5 Research Institute for Microbial Diseases, The University of Osaka, 3-1 Yamadaoka, Suita, Osaka 565-0871, Japan

**Keywords:** cefiderocol, carbapenemase-producing Enterobacterales, IMP-type carbapenemase, antimicrobial susceptibility, whole-genome sequencing, R2 loop of AmpC

## Abstract

**Objectives**: Carbapenem-resistant Enterobacterales (CRE) limit treatment options, and cefiderocol’s efficacy can be compromised by regional carbapenemases. In this study, we aimed to characterize cefiderocol susceptibility distribution among Enterobacterales in Japan—specifically endemic strains producing IMP-type carbapenemases (IMP)—and explore the effect of IMP in mediating resistance. **Methods**: The susceptibility of 556 evaluable Enterobacterales isolates (from an initial 560 screened) to cefiderocol was assessed using disk diffusion and broth microdilution methods based on Clinical and Laboratory Standards Institute (CLSI) guidelines. Minimum inhibitory concentration (MIC) distributions across groups were compared using Kruskal–Wallis and Mann–Whitney U tests. **Results**: A total of 556 isolates yielded evaluable results. The cefiderocol susceptibility rate was 99.5 and 98.7% based on CLSI and European Committee on Antimicrobial Susceptibility Testing breakpoints, respectively. Notably, under both criteria, IMP-producing isolates exhibited a susceptibility rate of 99.6%, whereas non-CRE and non-extended-spectrum β-lactamase (ESBL) exhibited a susceptibility rate of 99.4%. Statistical analysis revealed that the ESBL-only group had higher MICs than the non-CRE/non-ESBL and IMP-only groups (both *p* < 0.001), whereas no significant difference was observed between the latter two (*p* = 0.083). Notably, the ESBL-only group exhibited higher MICs than those of isolates harboring both IMP and ESBL. **Conclusions**: These findings indicate a non-additive effect, in which the coexistence of multiple resistance enzymes does not necessarily increase cefiderocol resistance. The association between IMP and cefiderocol resistance may be limited. In specific enzyme combinations, its presence was even associated with lower MICs.

## 1. Introduction

Antimicrobial resistance (AMR) has emerged as a global public health crisis, threatening the efficacy of modern medical treatments [1]. The global dissemination of extended-spectrum β-lactamase (ESBL) and carbapenemase-producing Enterobacterales (CPE) has compromised the efficacy of standard therapies, resulting in increased mortality and significant healthcare burden [2]. These resistance trends require effective therapeutic alternatives.

Cefiderocol is a novel siderophore cephalosporin designed to overcome common resistance mechanisms in Gram-negative bacteria, including β-lactamase production, porin mutations, and efflux pumps [3]. Structurally, it combines the features of cefepime and ceftazidime with a chlorocatechol moiety that enables siderophore-mediated iron transport into bacterial cells, thereby enhancing antibiotic uptake [4,5].

Cefiderocol demonstrates stability against carbapenemases, including metallo-β-lactamases, such as IMP-type carbapenemases (IMP), Verona integron-encoded metallo-β-lactamase (VIM), and New Delhi metallo-β-lactamase (NDM) [6]. However, resistance to cefiderocol has been associated with multiple mechanisms, including alterations in siderophore receptor genes, porin mutations, efflux pump activation, and modifications of penicillin-binding protein 3 (PBP3) [7]. Individual mechanisms alone are usually inadequate to confer clinical resistance—resistance commonly results from combinations of multiple factors [7]. Consequently, evaluating how these multi-layered genetic combinations influence real-world diagnostic performance is critical, particularly in regions where specific carbapenemase genotypes are heavily endemic.

Cefiderocol was first approved in the United States in 2019 for complicated urinary tract infections and later for hospital-acquired and ventilator-associated pneumonia [8]. In Europe, it was approved in 2020 for Gram-negative pathogen-caused infections with limited treatment options [9]. In Japan, cefiderocol was approved on 30 November 2023, for various infections caused by strains resistant to carbapenem antibiotics [10].

Globally, molecular and phenotypic surveillance of cefiderocol susceptibility has been extensively documented, particularly through large multinational programs in Europe and North America. In the SIDERO-WT surveillance studies, cefiderocol showed potent in vitro activity against a broad range of Gram-negative pathogens, including carbapenem-resistant isolates and carbapenemase producers, across more than 46,000 clinical isolates collected between 2014 and 2019 [11]. In Japan, large-scale surveillance studies provide an important epidemiologic baseline; however, there remains limited information on how specific co-existing resistance determinants affect categorical agreement between routine susceptibility methods and broth microdilution [12].

Surveillance data from the Japan Antimicrobial Resistant Bacterial Surveillance program reported cefiderocol susceptibility of 97.7 and 99.2% for CPE and IMP-type isolates, respectively [13]. However, the effect of IMP on cefiderocol susceptibility remains unclear. Additionally, susceptibility data for common clinical Enterobacterales isolates that are neither carbapenem-resistant Enterobacterales (CRE) nor ESBL producers remain limited in Japan.

To bridge this gap, the primary objective of this study was to characterize cefiderocol susceptibility and evaluate disk diffusion (DD) performance against a well-defined cohort of Enterobacterales clinical isolates collected in Osaka, Japan, across two distinct timelines (2020–2024 and 2015–2016). This regional focus provides precise, institutional diagnostic and therapeutic insights tailored for local nosocomial management.

## 2. Results

Among the 560 clinical isolates, four isolates were excluded because of inadequate growth during testing, leaving 556 evaluable isolates for final analysis.

### 2.1. Cefiderocol Susceptibility Among Enterobacterales Isolates

Using the DD method, six isolates were categorized as non-susceptible (Table 1); however, only two isolates (OU_24 and OU_186) were classified as non-susceptible by the broth microdilution (BMD) method. Additionally, one IMP-6-producing *Klebsiella aerogenes* isolate (OU_551) demonstrated discordant susceptibility results, being categorized as susceptible by the DD method but resistant by the BMD method.

### 2.2. Genomic Analysis of Cefiderocol Non-Susceptible Isolates

To assess the molecular mechanisms underlying reduced cefiderocol susceptibility, whole-genome sequencing (WGS) was performed for the three isolates identified as non-susceptible by the BMD method.

OU_24 was an ESBL-producing *E. coli* belonging to ST2011 and was categorized as intermediate by both DD and BMD methods. WGS analysis identified the β-lactamase gene (*bla*_CTX-M-55_), with no mutations or deletions observed in PBP3 or colicin I receptor A (*cirA*). However, multiple amino acid insertions were identified in outer membrane protein C (*ompC*) and outer membrane protein F (*ompF*), potentially altering the outer membrane permeability.

OU_186 was identified as *Enterobacter bugandensis* and classified as a non-CRE/non-ESBL AmpC β-lactamase (AmpC)-producing isolate. Multi-locus sequence typing (MLST) analysis revealed a novel sequence type (ST3387) that was newly assigned by the PubMLST database. OU_186 was categorized as resistant by both the DD and BMD methods. Genomic analysis identified the presence of *bla*_ACT-77_, Leu313 deletion (ΔL313) in the R2 loop of the AmpC, *cirA* gene deletion, and specific amino acid substitution (Y432F) in PBP3.

OU_551 was an ESBL- and IMP-producing *Klebsiella aerogenes* belonging to ST44231. The isolate carried *bla*_CTX-M-2_, *bla*_IMP-6_, and *bla*_AmpC-Kaer-1_ genes. Although no deletion was observed in the R2 loop of AmpC, the isolate exhibited deletions in *cirA* and *fiu*, along with three amino acid substitutions in PBP3 (S261T, A290S, and I563V).

### 2.3. Cefiderocol Susceptibility Rates

Cefiderocol exhibited potent antibacterial activity across different breakpoints. For the 556 evaluable isolates, susceptibility was 99.5% (553/556) using Clinical and Laboratory Standards Institute (CLSI) and 98.7% (549/556) using European Committee on Antimicrobial Susceptibility Testing (EUCAST) standards. Among the CPE isolates, the susceptibility rate of IMP-producing isolates reached 99.6% (258/259) based on CLSI or EUCAST. The susceptibility observed in the non-CRE/non-ESBL group was 99.4% (156/157) based on CLSI or EUCAST standards.

These results indicate that while IMP exert a minimal impact, ESBL production elevates cefiderocol MICs in this clinical isolate collection. However, this phenotypic elevation rarely caused the isolates to exceed the clinical resistance breakpoints, meaning cefiderocol retains strong overall efficacy.

## 3. Discussion

### 3.1. Analysis of Resistance Mechanisms

In the Kinki region of Japan, epidemiological surveillance indicates that *bla*_IMP_ variants remain the overwhelmingly predominant carbapenemase genes among Enterobacterales [14]. Our data reinforce this baseline, showing that while IMP production remains a formidable challenge for traditional β-lactams, cefiderocol maintains a high susceptibility rate against these MBL producers. The baseline cefiderocol susceptibility remained remarkably high and statistically indistinguishable between IMP-producing and non-IMP-producing isolates.

β-lactamases, AmpC enzymes, efflux pumps, or PBP3 mutations alone are generally inadequate to confer cefiderocol resistance [7]. Instead, resistance commonly emerges from multifactorial mechanisms involving concurrent alterations in iron transport systems, outer membrane permeability, and enzymatic activity [7]. Our findings align with previously reported data, indicating that reduced susceptibility to cefiderocol is likely a cumulative effect of complex, multifactorial resistance mechanisms.

To date, although the Phe313Leu mutation within the R2 loop has been reported to contribute to ceftazidime hydrolysis [15], there is no direct experimental evidence explicitly demonstrating that the Leu313 deletion (ΔL313) in AmpC independently confers resistance to cefiderocol. However, the structural integrity of the R2 loop in AmpC enzymes plays a critical role in substrate recognition. Amino acid deletions or mutations within the R2 loop of AmpC can result in reduced susceptibility to cefiderocol and other advanced cephalosporins [16,17]. Structural and functional studies showed that an R2-loop deletion increased the hydrolysis of cefiderocol and reduced susceptibility to ceftazidime-avibactam [16]. Furthermore, in a clinical setting reported that cefepime exposure selected for an R2-loop deletion that caused cefepime resistance and reduced cefiderocol susceptibility [17]. In this context, the available evidence is consistent with a potential role of the ΔL313 alteration in modulating cefiderocol activity; however, it does not by itself establish causality. The high-level resistance phenotype observed in *Enterobacter bugandensis* OU_186 is, therefore, more plausibly explained by the combined effects of multiple resistance mechanisms, including the ΔL313-associated PBP3 alteration and *cirA* deletion, rather than by any single determinant alone.

*Enterobacter bugandensis* is an increasingly recognized member of the *Enterobacter cloacae* complex, and previous reports have linked this species to severe neonatal infections and enhanced virulence in experimental models [18]. More broadly, *E. cloacae* complex organisms display substantial genomic diversity and a strong capacity to acquire resistance determinants, including β-lactamases and permeability-associated mechanisms [19,20]. Although *E. bugandensis* is not currently the most frequently recovered species in routine surveillance at our institution, its identification in the present study is microbiologically noteworthy.

### 3.2. Agreement Between DD and BMD Methods

**Figure 1:**
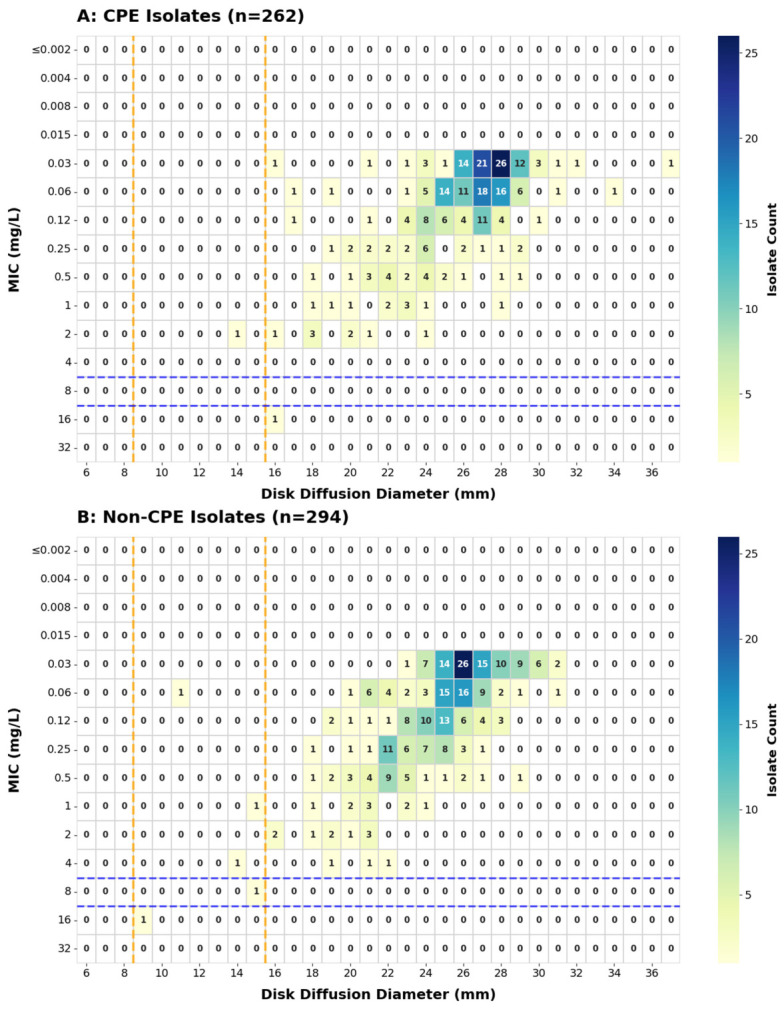
Correlation and distribution matrices of cefiderocol MICs and disk diffusion zone diameters. Heatmap matrices illustrate the two-dimensional distribution and correlation between cefiderocol MICs (mg/L) determined by broth microdilution and disk diffusion inhibition zone diameters (mm) across clinical isolates. (**A**) CPE isolates (n = 262). (**B**) Non-CPE Enterobacterales isolates (n = 294). Color intensity gradients represent the specific isolate counts within each coordinate, with cells containing zero isolates displayed in white. The vertical dashed orange lines flanking the 9–15 mm zone and horizontal dashed blue lines flanking the value of 8 mg/L indicate the provisional or established clinical breakpoints for susceptibility categorization.

Minimum inhibitory concentration (MIC) determination using the BMD method requires more specialized techniques than those of the DD method, which remains a more practical method for routine susceptibility testing [21,22]. The agreement between the DD and BMD methods for cefiderocol susceptibility testing was assessed using heatmap correlation analysis (Figure 1). Our research indicated a moderate-to-strong inverse correlation between the DD zone diameter and MIC values (Spearman r = −0.64, *p* < 0.001), supporting the use of cefiderocol DD testing as a reliable screening method—specifically for the rapid management of infections.

However, minor discrepancies and critical categorical errors were observed when inhibition zone diameters were close to the susceptibility breakpoint. Some isolates exhibited resistant MICs by BMD but were categorized as susceptible by DD, raising concern for potential very major errors (VMEs). A representative example was isolate OU_551, identified as *Klebsiella aerogenes*, which showed a discordant cefiderocol susceptibility result: the isolate was interpreted as susceptible by DD, with an inhibition zone diameter of 16 mm, whereas BMD demonstrated high-level resistance, with a cefiderocol MIC of 16 μg/mL. Molecular characterization revealed that OU_551 co-harbored *bla*_IMP-6_ together with ESBL and AmpC determinants.

IMP-6 is a well-characterized IMP variant in Japan; it differs from IMP-1 by a single amino acid substitution, S214G, and is associated with an altered substrate profile characterized by more efficient hydrolysis of meropenem than imipenem [23]. This biochemical feature may result in a clinically important “stealth” phenotype, in which carbapenemase-producing isolates retain relatively low MICs [24,25]. In the present isolate, the coexistence of IMP-6, ESBL, and AmpC enzymes may have contributed to reduced cefiderocol activity; however, the precise contribution of each β-lactamase to the cefiderocol-resistant phenotype cannot be determined from genotype alone.

The under-calling of cefiderocol resistance by DD in this genetic background has important diagnostic and therapeutic implications [26]. Misclassification of a phenotypically resistant isolate as susceptible could lead to inappropriate cefiderocol use and potential clinical failure. A plausible explanation for this DD–BMD discordance is the particular methodological sensitivity of cefiderocol susceptibility testing. Cefiderocol activity depends on bacterial iron-transport pathways, and MIC determination by BMD requires iron-depleted cation-adjusted Mueller–Hinton broth to approximate iron-restricted in vivo conditions [27,28]. By contrast, DD is performed on routine Mueller–Hinton agar rather than iron-depleted agar, and local cefiderocol diffusion, agar composition, iron availability, and zone-edge interpretation may influence categorical assignment, especially for isolates with inhibition zones near the breakpoint [27,28,29]. Despite these rare but critical baseline discrepancies, the two methods demonstrated a high categorical agreement (CA) for most isolates. Accounting for all discordant results, the CA between the DD and BMD methods was 98.9% (550/556). This indicates that DD testing still provides a highly reliable screening method for cefiderocol susceptibility with a minimal risk of overall misclassification. However, for isolates with inhibition zones close to the breakpoint, especially CRE, confirmatory MIC testing should be performed to minimize the risk of false susceptibility assignment [30,31,32].

### 3.3. Activity Against IMP-Producing Isolates

For presentation purposes, the bacterial isolates were definitively stratified into groups:

Non-CRE/non-ESBL: Isolates negative for both carbapenemase and ESBL production. ESBL-only: Isolates positive for ESBL but negative for carbapenemase.

IMP-only: Isolates positive for IMP but negative for ESBL.

IMP + ESBL: Isolates co-harboring IMP and producing ESBL.

In this study, the susceptibility rate of IMP-producing isolates was highly consistent with that observed among non-CRE/non-ESBL isolates. Similar findings have been reported previously, with studies demonstrating susceptibility rates over 99% among IMP-producing Enterobacterales [13,33,34], indicating that cefiderocol retains excellent activity against IMP [35].

**Figure 2:**
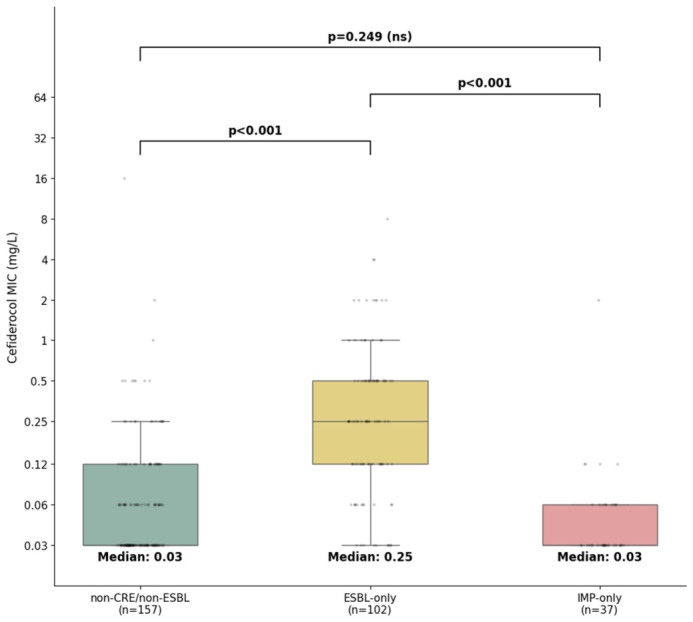
Distribution of cefiderocol MICs among non-CRE/non-ESBL, ESBL-only, and imipenemase-only isolates. Boxplots indicate the median and interquartile range, with whiskers extending to 1.5 times the interquartile range. Individual isolates are overlaid as jittered points. MIC values across groups exhibited a significant overall difference (*p* < 0.001). *p*-values were calculated using the Mann–Whitney U test and adjusted using the Bonferroni correction for multiple pairwise comparisons (ns, not significant). Median MIC values are annotated on each box. Abbreviations: non-CRE/non-ESBL, isolates without ESBL and CRE classification; ESBL-only, isolates with ESBL and without CRE classification; IMP-only, isolates harboring an IMP-type carbapenemase without ESBL.

Based on the statistical analysis, cefiderocol MICs differed significantly among non-CRE/non-ESBL isolates (n = 157), ESBL-only isolates (n = 102), and IMP-only isolates (n = 37) (overall *p* < 0.001; Figure 2), indicating that the enzyme type affects cefiderocol MIC distributions. However, MICs were significantly higher in the ESBL-only group than in the non-CRE/non-ESBL and IMP-only groups (both *p* < 0.001). In contrast, the difference between the non-CRE/non-ESBL and IMP-only groups was not significant (*p* = 0.083), indicating that IMP enzymes provide minimal protection against cefiderocol.

These results indicate that although ESBL production is associated with reduced cefiderocol susceptibility, IMP exert a limited effect on cefiderocol resistance.

### 3.4. Non-Additive Effect of Cefiderocol

**Figure 3:**
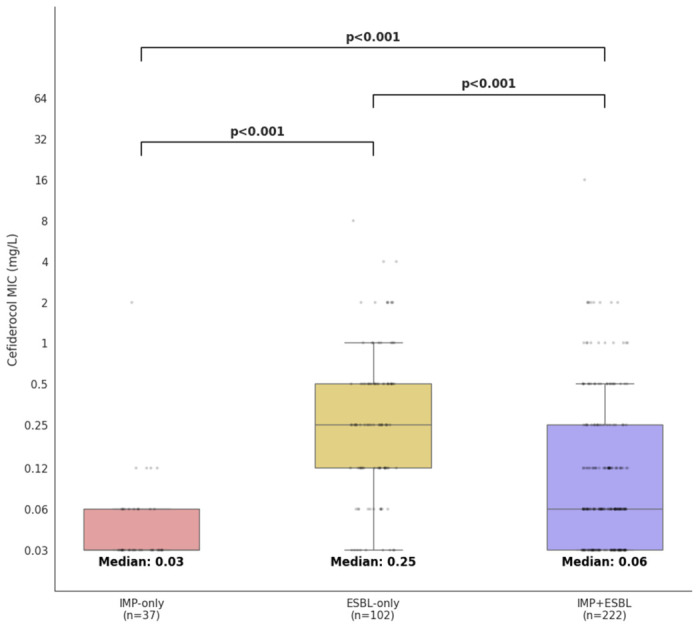
Distribution of cefiderocol MICs among IMP-only, ESBL-only, and IMP + ESBL isolates. Boxplots indicate the median and interquartile range, with whiskers extending to 1.5 times the interquartile range. Individual isolates are overlaid as jittered points. MIC values compared across groups exhibited a significant overall difference (*p* < 0.001), Pairwise statistical significance was determined by the Mann–Whitney U test with Bonferroni correction to control for Type I error inflation. Median MIC values are indicated next to each box plot. Abbreviations: IMP-only, isolates harboring an IMP-type carbapenemase without ESBL; ESBL-only, isolates with ESBL but without an IMP-type carbapenemase; IMP + ESBL, isolates harboring both an IMP-type carbapenemase and ESBL.

Recently, bacterial resistance to cefiderocol has involved the synergistic and additive effects of multiple mechanisms [36,37]. However, an interesting observation from this study was the non-additive effect between resistance mechanisms and cefiderocol activity. Cefiderocol MICs differed among IMP-only (n = 37), ESBL-only (n = 102), and IMP + ESBL (n = 222) isolates (overall *p* < 0.001; Figure 3), with pairwise comparisons exhibiting significant differences (all *p* < 0.001). The ESBL-only group exhibited a higher median MIC than that of the IMP + ESBL group, despite carrying fewer resistance determinants. This phenomenon indicates a non-additive interaction between the resistance mechanisms. The presence of specific enzymes, such as IMP, does not produce a cumulative increase in resistance and may, in specific combinations, be associated with reduced MICs.

One plausible explanation is that cefiderocol susceptibility in these isolates reflects a non-additive interaction between resistance burden, bacterial physiology, and drug-uptake pathways rather than a simple sum of β-lactamase activities. Plasmid-mediated resistance typically imposes significant fitness costs, driving compensatory evolution that subsequently remodels bacterial physiology [38]. Furthermore, antimicrobial resistance (AMR) epistasis dictates that the combined phenotype of multiple resistance determinants can deviate substantially from additivity. In that context, the lower cefiderocol MICs in the IMP + ESBL group may reflect a background-dependent epistatic trade-off: co-carriage of IMP and ESBL genes may alter plasmid burden, expression balance, or envelope homeostasis in a way that partially offsets β-lactamase-mediated resistance, paradoxically resulting in lower baseline MICs compared to the ESBL-only background [39].

Nonetheless, these observed variations in MIC distributions across the genomic groups may also be fundamentally influenced by other epidemiological confounding factors. As only a small number of isolates in our cohort exhibited phenotypic resistance to cefiderocol, these multi-layered molecular interaction remain just initial hypothetical frameworks that require rigorous functional validation in future genetic and biochemical studies.

### 3.5. Limitations and Future Perspectives

This study has some limitations that warrant consideration. First, the clinical isolates were collected from restricted geographical regions and specific facilities, which may limit the direct generalizability of our findings to the entirety of Japan. Second, a distinct temporal gap exists between the two collection cohorts (2015–2016 for the Osaka Institute of Public Health vs. 2020–2024 for The University of Osaka Hospital). While this decade-long separation could theoretically mask recent subtle shifts in regional molecular epidemiology or clonal lineage distributions, it is unlikely to compromise our core methodological conclusions, as the biochemical interactions between stable β-lactamase phenotypes and cefiderocol remain consistent regardless of the isolation year. Third, because WGS was selectively performed on only three non-susceptible strains to dissect outlier phenotypes, we could not account for the broader background resistome or other unmeasured resistance determinants across the rest of the cohort. Lastly, variations in underlying bacterial species between the ESBL-only and IMP + ESBL groups may have introduced unexpected confounding effects into the overall MIC distributions. Consequently, future prospective multi-center studies integrating larger, cohort-wide genomic datasets, plasmid copy-number determinations, and functional transcriptomic or membrane permeability analyses are strictly warranted to fully clarify the dynamic mechanisms underlying evolving cefiderocol resistance and to continuously optimize clinical susceptibility testing strategies.

## 4. Materials and Methods

### 4.1. Bacterial Isolates

Isolate Enrollment and Chronological Framework: To minimize selection bias, we initially targeted all consecutive, non-duplicate clinical isolates of Enterobacterales recovered at Osaka University Hospital during a contemporary period (March 2020 to October 2024), yielding 302 isolates. However, the baseline prevalence of CRE isolated purely within our facility was insufficient to provide adequate statistical power for robust molecular stratification. To address this sample limitation and establish a statistically viable evaluation platform, we strategically supplemented our collection with 258 distinct CRE clinical isolates consecutively referred to the Osaka Institute of Public Health during an earlier defined timeframe (November 2015 to January 2016).

In total, 560 Enterobacterales clinical isolates were initially screened across these two complementary cohorts. All isolates were definitively identified at the species level using matrix-assisted laser desorption/ionization–time-of-flight mass spectrometry (MALDI-TOF MS) via a MALDI Biotyper system (Bruker Daltonics, Bremen, Germany). Of the initial 560 isolates, four were excluded because of growth failure in subsequent susceptibility testing, leaving 556 isolates included in the final analysis.

Among Enterobacterales isolates, CRE was defined as isolates resistant to meropenem or producing carbapenemases. Carbapenemase production was characterized using the modified carbapenem inactivation method and the NG-Test CARBA 5 (NG Biotech, Guipry, France). Specifically, carbapenemase-producing Enterobacterales were categorized as CPE, whereas those without detectable carbapenemases were classified as non-CPE. A comprehensive antimicrobial susceptibility testing panel (Beckman Coulter, Brea, CA, USA) was utilized, which included meropenem (MEM), imipenem (IPM), ceftazidime (CAZ), cefotaxime (CTX), ceftriaxone (CRO), cefepime (FEP), and aztreonam (ATM).

**Figure 4:**
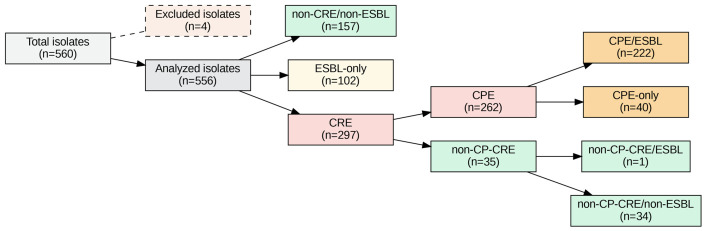
Flowchart of isolate distribution. A total of 556 Enterobacterales isolates are classified based on their CRE and ESBL phenotypes, and their CPE genotypes. Note: Four isolates from the initial collection are excluded from this flowchart because of inadequate growth during testing. Abbreviations: CRE, carbapenem-resistant Enterobacterales; CPE, carbapenemase-producing Enterobacterales; CP, carbapenemase; ESBL, extended-spectrum β-lactamase.

The following procedures were used for the detection and classification of carbapenemase and ESBL production:

i. CPE Detection: Isolates exhibiting a MEM MIC of ≥0.5 mg/L were initially screened for carbapenemase production using the modified carbapenem inactivation method (mCIM). For mCIM-positive strains, the presence of carbapenemase activity—specifically covering IMP type, VIM type, NDM type, KPC type, and OXA-48 type enzymes—was further confirmed using the NG-Test CARBA 5 (NG Biotech, Guipry, France).
ii. ESBL Detection: ESBL production was characterized strictly according to the CLSI M100-Ed34 criteria [29]. Initial screening and characterization of the resistance phenotypes were based on the MIC criteria for a comprehensive panel of cephalosporins and monobactams, followed by phenotypic confirmatory testing using combined disks (Becton Dickinson, Franklin Lakes, NJ, USA).
iii. IMP Group Classification: *bla*_IMP_ (n = 259) was the predominant genotype in the CPE group—222 isolates co-harbored *bla*_IMP_ and ESBL genes (CPE/ESBL), whereas 37 isolates carried *bla*_IMP_ alone. The other three CPE isolates were also ESBL-negative, including one carrying *bla*_IMI-1_ and two carrying *bla*_GES-24_. These 40 ESBL-negative isolates constitute the “CPE-only” group shown in Figure 4.

### 4.2. Antimicrobial Susceptibility Testing

Previous studies have demonstrated a high consistency between cefiderocol DD zone diameters and MIC values under CLSI criteria [40]. However, discrepancies have been reported in certain CRE isolates [30,31], highlighting the need for methodological validation in specific resistance genotypes, such as IMP. Therefore, in this study, all isolates were tested using both the DD and BMD methods.

DD testing was performed using 30 μg cefiderocol disks (Mast Group Ltd., Bootle, Merseyside, UK) on unsupplemented Mueller–Hinton agar (BD BBL™, Becton, Dickinson and Company, Franklin Lakes, NJ, USA) with a 0.5 McFarland inoculum. Readings were performed after incubation at 35 ± 2 °C for 20 ± 2 h. For BMD testing, commercial cefiderocol panels from Shionogi & Co., Ltd. (Osaka, Japan) were used following the manufacturer’s protocol. The same 0.5 McFarland inoculum used for the DD method was used for the BMD panels.

The results were interpreted based on the CLSI M100 (34th edition) [29] and EUCAST version 16.0 breakpoints [41]. For comparability, CLSI breakpoints were used for the analysis because previous nationwide surveillance studies conducted by Japanese committees have consistently reported susceptibility rates based on the CLSI criteria [13,35]. Currently, the gold standard for cefiderocol susceptibility testing is BMD-based MIC determination using cation-adjusted iron-depleted Mueller–Hinton broth [42]. In this study, the BMD results were used as the definitive standard for categorizing susceptibility.

### 4.3. WGS

For isolates identified as cefiderocol non-susceptible by BMD, WGS was performed to confirm species identification and characterize the molecular basis of resistance. Genomic DNA libraries were prepared using the Illumina DNA PCR-Free Prep kit and sequenced on the Illumina MiSeq platform (Illumina, San Diego, CA, USA) with 251-bp paired-end reads. Raw sequencing read quality was assessed using FastQC (v0.11.3). Subsequently, reads were assembled de novo using Unicycler (v0.4.4) [43] and annotated using the DFAST pipeline (v1.2.15) [44].

Antimicrobial resistance genes were identified using AMRFinderPlus (v4.0.23) [45] and the Comprehensive Antibiotic Resistance Database (CARD v4.0.1; RGI v6.0.5) [46]. Beyond the identification of β-lactamase genes, we assessed genetic alterations in three pathways: (i) iron uptake systems, including deletions or mutations in genes encoding catecholate siderophore receptors (*cirA* and *fiu*); (ii) target site modifications, focusing on amino acid substitutions or insertions in PBP3; and (iii) outer membrane permeability, by assessing structural variations in porin-encoding genes (*ompC* and *ompF*). Sequence alignments were performed against reference wild-type strains to identify specific mutations. MLST was determined using the PubMLST database [47], including the assignment of novel sequence types.

### 4.4. Statistical Analysis

The correlation between DD zone diameters and MIC values was assessed using Spearman’s rank correlation coefficient. The MIC distributions were compared among groups using the Kruskal–Wallis test, and post hoc pairwise comparisons were performed using the Mann–Whitney U test with Bonferroni correction to adjust for multiple comparisons. Adjusted *p*-values were calculated by multiplying the raw *p*-values by the number of total pairwise comparisons within each experiment (n = 3); a threshold of *p* < 0.05 was considered statistically significant.

All statistical analyses and data visualizations were implemented in Python (version 3.12.13). Specifically, the SciPy library (version 1.16.3) was used for statistical testing, whereas Pandas (version 2.2.2) and Matplotlib (version 3.10.0) were used for data management and visualization.

## 5. Conclusions

This study provides comprehensive baseline data on cefiderocol susceptibility among Enterobacterales clinical isolates in Japan, specifically focusing on those producing IMP. Our findings demonstrate that cefiderocol exhibits potent in vitro activity against IMP-producing strains and confirm a high CA (98.9%) between the routine DD and reference BMD methods. Notably, our statistical and genomic analyses highlight the highly complex, multifactorial nature of reduced cefiderocol susceptibility, as underscored by the unique non-additive effect observed in IMP + ESBL co-carriage isolates and the multi-layered molecular synergy involving AmpC mutations and receptor loss. These insights support the clinical utility of DD testing for routine cefiderocol surveillance while emphasizing that phenotypic outcomes are governed by dynamic physiological and genomic counterbalances rather than the simple accumulation of β-lactamase activities.

In conclusion, our evaluation of regional clinical isolates from Osaka demonstrates that cefiderocol retains high in vitro activity against carbapenemase-producing Enterobacterales. These findings establish a regional baseline that should be interpreted as an initial hypothesis warranting future multi-center validation across Japan.

## Figures and Tables

**Table 1:** Isolates demonstrating non-susceptibility to cefiderocol.

Isolate ID	Species	β-Lactamases	DD Method		BMD Method	
			Zone Diameter (mm)	Category	Minimum Inhibitory Concentration (μg/mL)	Category
OU_24	*Escherichia coli*	ESBL	15	I ^1^	8	I
OU_38	*Klebsiella pneumoniae*	ESBL	14	I	4	S ^2^
OU_46	*Klebsiella pneumoniae*	ESBL	15	I	1	S
OU_91	*Proteus mirabilis*	ESBL	11	I	0.06	S
OU_136	*Enterobacter cloacae*	IMP ^3^	14	I	2	S
OU_186	*Enterobacter bugandensis*	AmpC ^4^	9	I	16	R ^5^
OU_551	*Klebsiella aerogenes*	IMP + ESBL + AmpC	16	S	16	R

^1^ Intermediate. ^2^ Susceptible. ^3^ IMP-type carbapenemases. ^4^ AmpC β-lactamase. ^5^ Resistant.

## Data Availability

The datasets supporting the findings of this study are publicly archived in the FigShare repository. The whole-genome sequencing (WGS) data and antimicrobial susceptibility test results can be accessed via the Digital Object Identifier (DOI): 10.6084/m9.figshare.32446335.
